# ENGDM: Enhanced Non-Isotropic Gaussian Diffusion Model for Progressive Image Editing †

**DOI:** 10.3390/s25102970

**Published:** 2025-05-08

**Authors:** Xi Yu, Xiang Gu, Xin Hu, Jian Sun

**Affiliations:** School of Mathematics and Statistics, Xi’an Jiaotong University, Xi’an 710049, China; ericayu@stu.xjtu.edu.cn (X.Y.); xianggu@stu.xjtu.edu.cn (X.G.); huxin7020@stu.xjtu.edu.cn (X.H.)

**Keywords:** diffusion models, progressive image editing, enhanced non-isotropic Gaussian diffusion model

## Abstract

Diffusion models have made remarkable progress in image generation, leading to advancements in the field of image editing. However, balancing editability with faithfulness remains a significant challenge. Motivated by the fact that more novel content will be generated when larger variance noise is applied to the image, in this paper, we propose an Enhanced Non-isotropic Gaussian Diffusion Model (ENGDM) for progressive image editing, which introduces independent Gaussian noise with varying variances to each pixel based on its editing needs. To enable efficient inference without retraining, ENGDM is rectified into an isotropic Gaussian diffusion model (IGDM) by assigning different total diffusion times to different pixels. Furthermore, we introduce reinforced text embeddings, using a novel editing reinforcement loss in the latent space to optimize text embeddings for enhanced editability. And we introduce optimized noise variances by employing a structural consistency loss to dynamically adjust the denoising time steps for each pixel for better faithfulness. Experimental results on multiple datasets demonstrate that ENGDM achieves state-of-the-art performance in image-editing tasks, effectively balancing faithfulness to the source image and alignment with the desired editing target.

## 1. Introduction

Image editing [1,2,3,4,5] has attracted extensive attention in recent years, thanks to the development of diffusion models [6,7,8,9,10,11]. This task aims to edit a source image into a target image based on a given target text prompt while preserving high faithfulness to the source image in regions unrelated to the editing task.

**Figure 1:**
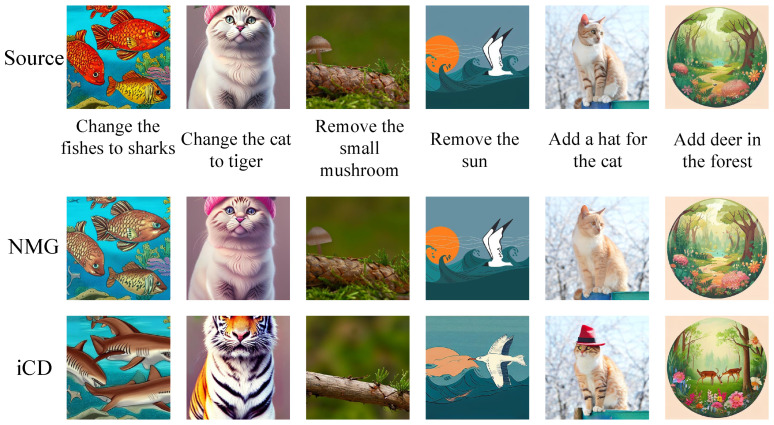
Concrete examples illustrating the challenge in achieving a balance between editability and faithfulness.

Existing image-editing methods are primarily classified into three categories: mask based, inversion based, and attention based. Mask-based methods [4,12,13,14] leverage masks to guide the sampling process, ensuring that editing is performed only in the mask region. Inversion-based methods [3,15,16,17,18,19] map real images to noisy latents through an inversion process and then use a sampling process to generate edited images based on the given target text. Attention-based methods [2,20,21,22] modify the attention layers of the U-Net network in Stable Diffusion models to preserve the features of the source image. While these approaches have significantly advanced the capabilities of image editing, achieving an optimal balance between editability and faithfulness remains a challenge. For example, as shown in Figure 1, NMG [19] applies insufficient editing, failing to achieve the desired editing task. In contrast, iCD [23] tends to over-edit, resulting in significant deviations between the edited and source images.

To address these challenges, we propose an Enhanced Non-isotropic Gaussian Diffusion Model (ENGDM) for progressive image editing, which is extended from our conference paper NGDM [24] published in NeurIPS. Diffusion models are empirically known to generate more diverse and novel content when larger variance noise is added, while preserving the image content when smaller variance noise is applied [1]. Motivated by this, we employ a non-isotropic Gaussian diffusion model (NGDM) to add independent Gaussian noises with different variances to different image pixels. The variance is determined by the degree to which each pixel needs to be edited, allowing precise control over the editing process. To avoid retraining the score model for ENGDM, we rectify the NGDM to implement ENGDM within the framework of IGDM. Each pixel is added an equal amount of noise at each step, but different pixels accumulate noise over varying total time steps.

To further enhance the editability of the model, we incorporate reinforced text embeddings during the sampling process. We design a novel editing reinforcement loss operating in the latent space to optimize the text embeddings, guiding the model to accurately generate the target objects specified in the textual description. Meanwhile, to maintain high faithfulness to the source image, we optimize noise variances by using a structural consistency loss to dynamically adjust the denoising time steps for each pixel, ensuring the preservation of fine-grained details of the source image. Lastly, we design a specialized sampling strategy that seamlessly integrates ENGDM with a pre-trained IGDM (e.g., Stable Diffusion [25]), enabling high-quality image editing.

Our contributions are summarized as follows:

- We propose ENGDM, a novel method for progressive image editing. We introduce reinforced text embeddings, using a novel editing reinforcement loss in the latent space to optimize text embeddings for enhanced editability.
- We propose the optimized noise variances by employing a structural consistency loss to dynamically adjust the denoising time steps for each pixel, ensuring high faithfulness to the source image.
- Extensive experiments on multiple datasets demonstrate that ENGDM achieves state-of-the-art performance in image-editing tasks, achieving a better balance between editability and faithfulness.

This paper extends our conference version NGDM [24] published in NeurIPS, in which we devised the non-isotropic Gaussian diffusion process for image editing. In this journal version, we make additional contributions with reinforced text embeddings, optimized noise variances, a refined sampling algorithm, and further performance improvements.

The rest of the paper is structured as follows. We summarize the related works in Section 2. Section 3 presents the background of the Gaussian diffusion model. Section 4 introduces our ENGDM method. Section 5 discusses the experimental results. Section 6 concludes this paper.

## 2. Related Work

Image editing aims to modify a user-provided source image to align with a given target prompt while minimizing visual changes to the source image. We summarize the related works in Table 1.

**Mask-based image editing.** Mask-based image-editing methods leverage masks to guide and refine the sampling process. These approaches enable models to focus on precisely modifying localized regions to align with the editing target. Blended Diffusion [13] and Blended Latent Diffusion [14] employ user-provided masks to blend the noisy latents from the forward noising process of the Denoising Diffusion Probabilistic Model (DDPM) [8] with the noisy latents from the denoising process, thereby restricting modifications to the specified regions. PFB-Diff [26] performs blending not directly on intermediate noisy latents but instead on feature maps. PFB-Diff seamlessly integrates generated content guided by the target prompt through multi-level feature blending and introduces an attention-masking mechanism in the cross-attention layers to improve editing performance. Without requiring user-provided masks, DiffEdit [4] automatically generates a mask by contrasting the predictions of a diffusion model conditioned on different text prompts, highlighting the regions in the source image that need to be edited. RDM [27] introduces a novel region generator model that employs a new CLIP-guided loss to learn how to identify the image-editing regions. These methods rely on predefined editing regions and use a fixed-size mask throughout the entire denoising process. In contrast, our approach progressively increases the editable regions during the denoising process, enabling progressive and dynamic image editing. This strategy effectively mitigates edge artifacts and enhances faithfulness to the source image, addressing the limitations of hard mask-guided approaches.

**Inversion-based image editing.** Inversion-based image-editing methods invert the real image into the initial noise, enabling the generation of edited results based on this initial point and a given target prompt. The pioneering research DDIM Inversion [28] proposes deterministic inversion with the discretization of diffusion ODE to encode the source image into noisy variables to preserve the source image information. However, under the classifier-free guidance, DDIM suffers from significant accumulated error, resulting in reconstruction failures. Subsequent works [3,18,19,29,30] mitigate the accumulated error by approximating the inversion trajectory. For instance, Null-Text Inversion [3] optimizes the null text embedding by minimizing the discrepancy between the ideal and actual intermediate latents, while PnP Inversion [18] introduces correction terms to achieve the same goal. Another group of works [17,31,32] adopt stochastic differential equation (SDE) instead of ODE, reducing errors by manipulating random noise. SDE-Drag [32] demonstrates that stochastic inversion outperforms deterministic inversion in editing performance, and the KL divergence between the distribution of edited image and prior data distribution decreases in stochastic inversion while remaining in deterministic inversion. Additionally, some works [16,33,34] establish mathematically exact inversion. EDICT [16] achieves mathematically exact inversion by tracking two noisy variables in each step during inversion, which can be derived from each other in the sampling time.

**Attention-based image editing.** The attention layers in the U-Net network of Stable Diffusion play a significant role in determining the layout of the generated image. Attention-based methods achieve image editing by manipulating the attention layers. P2P [2] demonstrates that the cross-attention maps govern the spatial layout and structure of the generated image. Accordingly, it preserves the structure of the source image by replacing the cross-attention maps during the denoising process with those from the reconstruction process. Inspired by P2P [2], subsequent works [35,36,37,38] leverage cross-attention maps for maintaining the structure of the source image. Several methods [20,22,39,40] perform image editing by modifying self-attention maps instead of cross-attention maps. FPE [22] highlights that modifying cross-attention maps may lead to editing failures, emphasizing that self-attention maps are crucial for the success of image-editing tasks. Beyond modifying attention maps, MasaCtrl [21] and PnP [20] enforce consistency between the edited and source images by directly modifying specific attention features.

Differently, our method performs progressive image editing by introducing independent Gaussian noises with varying variances to different pixels, guided by a weighting matrix with soft weights.

**Table 1:** Summarization of related works.

Editing Method	Mask-Based	Inversion-Based	Attention-Based
Study	Blended Diffusion [13], Blended Latent Diffusion [14], PFB-Diff [26], DiffEdit [4], RDM [27]	DDIM Inversion [28], Null-Text Inversion [3], PnP Inversion [18], NMG [19], PTI [29], ProxEdit [30], DDPM Inversion [17], LEDITS++ [31], SDE-Drag [32], EDICT [16], BELM [33]	P2P [2], Pix2Pix-Zero [35], Custom-edit [36], Conditional Score Guidance [37], PnP [20], FPE [22], Photoswap [39], StyleInjection [40], MasaCtrl [21]
Purpose	Mask-based image-editing methods leverage masks to guide and refine the sampling process.	Inversion-based image-editing methods invert the real image into noise space, and then use the sampling process to generate the edited results based on the noisy latent and a given target prompt.	Attention-based methods achieve image editing by manipulating the attention layers.
Limitation	The method exhibits limited flexibility when handling complex modifications.	The inversion process is time-consuming and may hinder practical applications.	It is challenging to accurately locate the specific regions that require editing.
Performance	The faithfulness of non-edited regions is high, but edge artifacts are prone to occur.	The details of the source image can be effectively preserved, but easy to fail editing in complex scenarios.	The details of the source image are not precisely preserved.

## 3. Background: Score-Based Diffusion Models

Diffusion models [6,7,8,9,10] are a family of generative models that learn the data distribution based on the Gaussian process. Two representative models are the Denoising Diffusion Probabilistic Model (DDPM) [8] and Score Matching with Langevin Dynamics (SMLD) [7]. We discuss the details based on DDPM for the remainder of the paper for brevity.

Given the input data $x\left(0\right)\in R^{D}$, which represents a sample from the data distribution $p_{data}$, a forward process produces the noisy x(t) indexed by a time variable t∈[0,1] via

(1) $$x\left(t\right)=\sqrt{\bar{\alpha }\left(t\right)}x\left(0\right)+\sqrt{1-\bar{\alpha }\left(t\right)}z\left(t\right),$$

where z(t)∈N(0,I) for any *t* and $\bar{\alpha }\left(t\right)=e^{-\int_{0}^{t}\beta \left(s\right)ds}$ controlling the noise schedule. $\beta \left(s\right)={\bar{\beta }}_{\min}+s\left({\bar{\beta }}_{\max}-{\bar{\beta }}_{\min}\right)$ with ${\bar{\beta }}_{\min}=0.1$ and ${\bar{\beta }}_{\max}=20$ [8,9]. This type of diffusion model is dubbed *IGDM* since the added Gaussian noise z(t) is from the independently and identically distributed normal distribution.

DDPM is in the framework of SDE [7] with variance preservation

(2) $$dx\left(t\right)=-\frac{1}{2}\beta \left(t\right)x\left(t\right)dt+\sqrt{\beta (t)}dw,$$

where w is the standard Wiener process and the initial value of the above SDE is x(0). The reverse process denoises the noisy sample x(T) starting from *T* using a reverse SDE

(3) $$\begin{array}{c} dx\left(t\right)=\left[-\frac{1}{2}\beta \left(t\right)x\left(t\right)-\beta \left(t\right)\nabla_{x}\log p_{t}\left(x\left(t\right)\right)\right]dt+\sqrt{\beta (t)}d\bar{w}, \end{array}$$

where $\bar{w}$ is a standard Wiener process when time flows backward from *T* to 0 and the initial value of the above SDE is x(T). The score function $\nabla_{x}\log p_{t}\left(x\right)$ is approximated by training a time-dependent model $s_{\theta }\left(x\left(t\right),t,C\right)$ under condition C via score matching [9,41]. For inference, the time of the differential equation is discretized as $t\in \left\{0,\Delta t,2\Delta t,\cdots ,T\right\}$, with Δt representing the sampling time interval. We choose to utilize the reverse process of DDIM for sampling. With $\beta_{t}=\beta \left(t\right)\Delta t$ and ${\bar{\alpha }}_{t}=\prod_{s=0}^{t}\left(1-\beta_{s}\right)$, the deterministic iteration rule of DDIM [28] is

(4) $$\begin{array}{c} x\left(t\right)=\sqrt{{\bar{\alpha }}_{t}}\hat{x}\left(0,t+\Delta t\right)+\sqrt{1-{\bar{\alpha }}_{t}}s_{\theta }\left(x\left(t+\Delta t\right),t+\Delta t,C\right), \end{array}$$

where $\hat{x}\left(0,t+\Delta t\right)$ is the prediction of the initial data point x(0) at time *t* and is derived as

(5) $$\begin{array}{c} \hat{x}\left(0,t+\Delta t\right)=\frac{x\left(t+\Delta t\right)-\sqrt{1-{\bar{\alpha }}_{t+\Delta t}}s_{\theta }\left(x\left(t+\Delta t\right),t+\Delta t,C\right)}{\sqrt{{\bar{\alpha }}_{t+\Delta t}}}. \end{array}$$

## 4. Method

**Figure 2:**
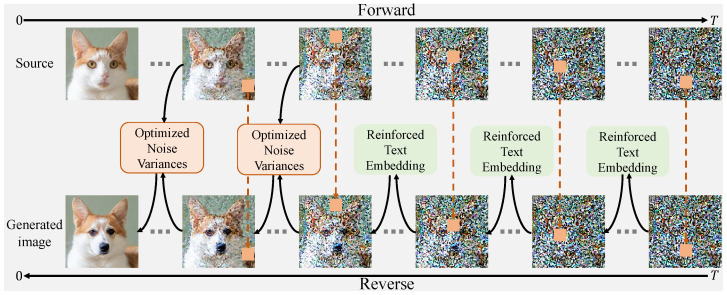
The overview of our ENGDM. Firstly, isotropic noises are added to the source image until *T* time steps. Then, in the reverse denoising process, different pixels begin the denoising process at different time steps as indicated by the orange blocks and lines. Additionally, in the early stages of denoising, the learned reinforced text embeddings are incorporated to enhance editability. In the later stages, the optimized noise variance is incorporated to improve fidelity.

In this section, we introduce the ENGDM for progressive image editing. We first construct the NGDM in Section 4.1, then we rectify the non-isotropic Gaussian diffusion process in Section 4.2. To further enhance the editability and faithfulness, we propose reinforced text embeddings in Section 4.3 and optimized noise variances in Section 4.4. Finally, we design a novel sampling algorithm in Section 4.5. The overview of ENGDM is shown in Figure 2.

### 4.1. Non-Isotropic Gaussian Diffusion Model

We construct the NGDM by adding the non-isotropic Gaussian noise in the input data $y\left(0\right)\in R^{D}$ and $y\left(0\right)∼p_{data}$, and the noises associated with different pixels are independent. The forward SDE of NGDM [24] is

(6) $$dy\left(t\right)=-\frac{1}{2}\beta \left(t\right)\Lambda \left(I\right)y\left(t\right)dt+\sqrt{\beta (t)\Lambda (I)}dw,$$

where $I\in R^{D}$ is the source data, $\Lambda \left(I\right):R^{D}\to R^{D\times D}$ is the weighting matrix, defined as diagonal matrix $\Lambda \left(I\right)=\mathrm{diag}\left(\lambda_{1},\cdots ,\lambda_{D}\right)$ with $0\leq \lambda_{k}\leq 1$ scaling the Gaussian noise level added to the *k*-th pixel. The initial value of the above SDE is y(0). Note that the transition kernel $p_{0t}\left(y\left(t\right)|y\left(0\right)\right)=N\left(y\left(t\right)|y\left(0\right)e^{-\frac{1}{2}\int_{0}^{t}\beta \left(s\right)\Lambda \left(I\right)ds},I-e^{-\int_{0}^{t}\beta \left(s\right)\Lambda \left(I\right)ds}\right)$ is an independent Gaussian distribution.

### 4.2. Rectify the Non-Isotropic Gaussian Diffusion Model

With the added independent noise, we next discuss the forward SDE for NGDM [24] in scalar form for each pixel *k*. Given the initial $y^{k}\left(0\right)$ denoting the value of pixel *k* in y(0), the forward SDE of the *k*-th pixel can be presented by

(7) $$dy^{k}\left(t\right)=-\frac{1}{2}\beta \left(t\right)\lambda_{k}y^{k}\left(t\right)dt+\sqrt{\beta \left(t\right)\lambda_{k}}dw,$$

where w is a one-dimensional Wiener process and the initial value of the SDE is $y^{k}\left(0\right)$.

We present Lemma 1 and Theorem 1 proposed in our conference version [24] to illustrate the connection between the NGDM defined in Section 4.1 and the IGDM defined in Section 3 at the pixel level.

**Lemma** **1.** *Let $\beta \left(s\right)={\bar{\beta }}_{\min}+s\left({\bar{\beta }}_{\max}-{\bar{\beta }}_{\min}\right)$, with ${\bar{\beta }}_{\max}>{\bar{\beta }}_{\min}>0$. Then, for each $\lambda_{k}\in \left[0,1\right]$ and t∈[0,1], there exists a unique time τ∈[0,1] (denoted by $\tau =\xi_{k}\left(t\right)$) such that $\int_{0}^{t}\beta \left(s\right)\lambda_{k}ds=\int_{0}^{\tau }\beta \left(s\right)ds$ and $\beta \left(t\right)\lambda_{k}dt=\beta \left(\tau \right)d\tau$, with the following form*

(8) $$\begin{array}{c} \xi_{k}\left(t\right)=\frac{-{\bar{\beta }}_{\min}}{{\bar{\beta }}_{\max}-{\bar{\beta }}_{\min}}+\sqrt{{\left(\frac{{\bar{\beta }}_{\min}}{{\bar{\beta }}_{\max}-{\bar{\beta }}_{\min}}\right)}^{2}+\frac{2{\bar{\beta }}_{\min}t\lambda_{k}}{{\bar{\beta }}_{\max}-{\bar{\beta }}_{\min}}+t^{2}\lambda_{k}}. \end{array}$$

Based on the above Lemma, we can rectify the NGDM, which adds noise at each pixel with varying variance over the same time span, into an IGDM that adds noise at each pixel with the same noise variance but with a different total diffusion time for different pixels. We introduce the following theorem to derive the differential equation as an IGDM.

**Theorem** **1.** *For a pixel indexed by k, $\lambda_{k}\in \left[0,1\right]$, and let $\tau =\xi_{k}\left(t\right)$ with $\xi_{k}\left(t\right)$ represented in Equation (8). With the same initial value $y^{k}\left(0\right)$, we have that the transition kernel at time t induced by Equation (7) is equal to the transition kernel at time τ induced by the following differential equation:*

(9) $$dy^{k}\left(t\right)=-\frac{1}{2}\beta \left(\tau \right)y^{k}\left(t\right)d\tau +\sqrt{\beta (\tau )}dw.$$

*The initial condition of the above SDE is $y^{k}\left(0\right)$. The total time of noising for Equation (9) is $T_{k}$ with $T_{k}=\xi_{k}\left(T\right)$.*

Inspired by this, we rectify the reverse process in NGDM with different speeds of denoising across pixels to be the reverse process with consistent speed but different total time of denoising. We propose rectifying the differential equation for the reverse process within the NGDM framework into the following form:

(10) $$dy^{k}\left(t\right)=\left[-\frac{1}{2}\beta \left(\tau \right)y^{k}\left(t\right)-\beta \left(\tau \right){\left(\nabla_{y}\log p_{\tau }\left(y\left(t\right)\right)\right)}^{k}\right]d\tau +\sqrt{\beta (\tau )}d\bar{w},$$

where $\bar{w}$ is a one-dimensional Wiener process when time flows backward from $T_{k}$ to 0, and the initial value of the above SDE is $y^{k}\left(T_{k}\right)$. Theorem 1 establishes the conclusion that the NGDM in Equation (7) can be rectified to the IGDM in Equation (9) but with different total diffusion time $T_{k}$ for different pixel indexed by *k*, determined based on Equation (8). This inspires us to utilize the pre-trained IGDM to achieve the data-sampling of NGDM for image editing. Subsequently, we propose adjusting the total time of noising and denoising for each pixel *k* to $T_{k}$, enabling the utilization of pre-trained IGDM for data sampling.

For image-editing tasks, we use the source image I as y(0) and generate noisy data y(T) through the forward process. We generate the edited image $\hat{y}\left(0\right)$ by denoising from y(T). Utilizing the forward noising process of IGDM, we add independent noise to each pixel *k* to obtain the noisy observation $x^{k}\left(t\right)$ of discrete time $t\in \left\{0,\Delta t,\cdots ,T\right\}$ with Δt representing the sampling time interval

(11) $$x^{k}\left(t\right)=\sqrt{{\bar{\alpha }}_{t}}I^{k}+\sqrt{1-{\bar{\alpha }}_{t}}z^{k}\left(t\right),$$

where z(t)∈N(0,I). Next, with H(y(t+Δt),t+Δt) denoting the sampling procedure of DDIM given in Equation (4) of Section 3, the data-sampling iteration utilizing the IGDM model with initial value $y^{k}\left(T\right)=x^{k}\left(T\right)$ is defined as

(12) $$\begin{array}{c} y^{k}\left(t\right)=M^{k}\left(t\right)⊙H^{k}\left(y\left(t+\Delta t\right),t+\Delta t\right)+\left(1-M^{k}\left(t\right)\right)⊙x^{k}\left(t\right), \end{array}$$

where $M^{k}\left(t\right)=I\left(t<T_{k}\right\})$. This implies that we use the noisy observation $x^{k}\left(t\right)$ to represent $y^{k}\left(t\right)$ at each step before $T_{k}$ with $t\geq T_{k}$, rather than the actual denoised result starting from time step *T*. Until time $T_{k}$, we begin the denoising from $x^{k}\left(T_{k}\right)$ for the *k*-th pixel. In such a way, different pixels have different starting time steps ($T_{k}$ for *k*-th pixel) for image denoising in the data-sampling process.

We next combine all pixels to perform the denoising process using the complete data. We introduce a time-dependent mask M(t) to control the denoising state of each pixel. Specifically, M(t) determines which pixels begin denoising at time step *t* and which remain unprocessed. The mask M(t) is defined based on the relationship between the denoising start time step $T_{k}$ of each pixel and the current time step *t*. Formally, M(t) can be defined as $M\left(t\right)=[m_{1}\left(t\right),\cdots ,m_{D}\left(t\right)]$ with

(13) $$m_{k}=\left\{\begin{array}{cc} 0 & \mathrm{if}\;T_{k}\leq t\\ 1 & \mathrm{if}\;T_{k}>t. \end{array}\right.$$

The *k*-th element of M(t), denoted $m_{k}\left(t\right)$, indicates the denoising state of the *k*-th pixel at time *t*. $m_{k}\left(t\right)=1$ means that the pixel has already started denoising at time step *t*, while $m_{k}\left(t\right)=0$ indicates that denoising has not yet started.

Based on the relationship between the noise variance and the time step given by Equation (8) in Lemma 1, we can compute the noise variance corresponding to the time step *t*, denoted as $\bar{\lambda }\left(t\right)$. Then, $m_{k}\left(t\right)$ can be equivalently represented as

(14) $$m_{k}\left(t\right)=\left\{\begin{array}{cc} 0 & \mathrm{if}\;\lambda_{k}\leq \bar{\lambda }\left(t\right)\\ 1 & \mathrm{if}\;\lambda_{k}>\bar{\lambda }\left(t\right). \end{array}\right.$$

Equipped with M(t), we can then obtain the iteration rule of complete noisy observation y(t) in the following form:

(15) $$\begin{array}{c} y(t)=M(t)⊙H(y(t+\Delta t),t+\Delta t)+(1-M(t))⊙x(t). \end{array}$$

The pixels of y(t) begin the denoising process at different time steps.

### 4.3. Enhancing Editability with Reinforced Text Embeddings

To enable sufficient editing, we incorporate the reinforced text embedding optimization strategy into the diffusion process. This strategy builds upon the classifier-free guidance technique [42], which removes the need for a separate classifier to guide diffusion models. Formally, let ⊘ be the embedding of a null text “ ” and let *w* be the guidance scale, then the classifier-free guidance prediction [42] is defined by

(16) $$\begin{array}{c} {\tilde{s}}_{\theta }\left(y\left(t\right),t,C\right)=s_{\theta }\left(y\left(t\right),t,⊘\right)+w\cdot s_{\theta }\left(y\left(t\right),t,C\right). \end{array}$$

The guidance scale *w* controls the degree of alignment between the generated image and the textual condition. A larger *w* increases the strength of the conditioning, improving the alignment with the text prompt but at the cost of the visual quality of the generated image.

We propose the reinforced text embedding optimization strategy to strengthen the editing intensity without sacrificing the visual quality of the generated image. The key idea behind the reinforced text embeddings is to optimize the text embeddings by using an optimization strategy that pulls the latent closer to the reinforced one. This reinforced latent is achieved by using a larger guidance scale, which increases the model’s focus on aligning the image with the text prompt. Instead of directly increasing the guidance scale, we optimize the text embeddings by pushing the latent closer to those under stronger guidance. The reinforced text embeddings C is optimized using the following editing reinforcement loss:

(17) $$L_{\mathrm{edit}}={∥y\left(t\right)-y^{\prime }\left(t\right)∥}_{2}^{2}.$$

The noisy latent y(t) at step *t* is obtained from Equation (15), which combines the denoised result H(y(t+Δt),t+Δt) with guidance scale *w* and the noisy observation x(t) from the forward process. The denoised result $y^{\prime }\left(t\right)$ at the same step is obtained using a larger guidance scale $w^{\prime }$ with $w^{\prime }>w$. Unlike some methods [27,43] that use CLIP loss [44] to align images and target text prompt, which makes the optimization process difficult and resource-consuming, our proposed editing reinforcement loss can be optimized directly in the latent space in an efficient way. The loss function for optimizing the text embeddings is applied only during the early stages of the denoising process, as these stages are more inclined toward generating diverse content.

By aligning y(t) with the denoised results produced under stronger guidance conditions, the model learns reinforced text embeddings, which effectively enhance its editing capabilities. This approach not only enhances the editing performance of the model but also mitigates the need for excessively high guidance scales, thereby mitigating potential visual artifacts.

### 4.4. Enhancing Faithfulness with Optimized Noise Variances

Our method relies on the weighting matrix Λ(I) defined in NGDM [24], which determines the extent of the noise variance applied to each pixel based on the editing requirements. We first initialize the weighting matrix A(I) based on DiffEdit [4]. Following DiffEdit, we determine the editing degree of each pixel by analyzing the differences between score estimates produced by the diffusion model under different text conditions. Specifically, given the source image I, the source prompt *R* of the source image, and the target prompt *Q* that describes the desired target image after editing, we add noise to the source image up to the 0.5T step and use the texts *R* and *Q* as the conditions, respectively, for denoising in the current time step to estimate the score values by using the score network $s_{\theta }$. We derive the degree map of editing based on the absolute difference of the estimated scores. We use the above method to compute 10 estimated absolute noise differences by running 10 times with different random seeds, averaging and performing Gaussian smoothing on the averaged map to obtain the final degree map A(I).

The pixel with the larger value in the degree map should be added with the noise with larger variance. We define the weighting matrix Λ(I) by applying a sigmoid function to the degree map A(I), i.e., $\Lambda \left(I\right)=\frac{1}{1+\exp(aA(I)+b)}$, with *a* and *b* denoting the hyperparameters for the transformation. By substituting each pixel’s noise variance and the maximum diffusion time step *T* into Equation (8), we can obtain the total diffusion time for each pixel. We discuss the effect of hyperparameters *a* and *b* on the generation of images in Section 5.3.2 and validate the effectiveness of the method for determining the total diffusion time step of each pixel in Section 5.3.5.

Precise editing relies on accurate estimation of Λ(I). In practice, we observe that editing failures are most commonly caused by assigning excessively large variances to pixels that do not require editing, leading to unnecessary modifications in non-edit regions, thus reducing the faithfulness of the generated image. To address this limitation, we further propose an optimization-based approach to refine Λ(I), dynamically correcting the weighting matrix in the later stages of the diffusion process. To obtain the denoised result of step *t*, we use $\Lambda^{*}\left(I\right)$ and Equation (13) to determine the mask M(t) for the current time step, and then use Equation (15) to obtain the updated denoising result y(t). We optimize $\Lambda^{*}\left(I\right)$ by constraining the structural similarity between the denoising result H(y(t+Δt),t+Δt) and the noisy observation x(t) in the forward process. To enable gradient-based optimization, we approximate the mask M(t) used in Equation (15) with a sigmoid function. We minimize the mean squared error (MSE) between the structural similarity of the edited and source images to optimize $\Lambda^{*}\left(I\right)$. The optimization objective is

(18) $$\begin{array}{c} L_{\mathrm{faithful}}={∥S\left(H\left(y\left(t+\Delta t\right),t+\Delta t\right)\right)-S\left(x\left(t\right)\right)∥}_{2}^{2}+\rho {∥\Lambda^{*}\left(I\right)-\Lambda \left(I\right)∥}_{2}^{2}, \end{array}$$

where S(·) represents the structure self-similarity [45] function with ${\left[S\left(z\right)\right]}_{i,j}=\frac{z_{i}z_{j}^{T}}{∥z_{i}∥∥z_{j}∥}$, *i* and *j* are the pixel indices. The $∥\Lambda^{*}{\left(I\right)-\Lambda \left(I\right)∥}_{2}^{2}$ is the regularization term, and ρ is the coefficient of the regularization term.

### 4.5. Sampling Method in ENGDM

Based on the above method, we specify our sampling algorithm by harnessing the power of a pre-trained IGDM. We generate the edited image with the source image I as a condition. By utilizing DDIM inversion, we first add noise to the source image I to *T* time steps, and then use the method in Section 4.2 to rectify NGDM into IGDM to denoise the image. Additionally, we learn reinforced text embeddings and optimized noise variances. We show the sampling algorithm of ENGDM in Algorithm 1.
**Algorithm 1** Sampling method of ENGDM.**Inputs:** The source image I, the time schedule ${\left\{\beta \left(t\right)\right\}}_{t=0}^{T}$, the maximal time steps *T*, the optimization time $T_{s}$, the guidance scale *w* and $w^{\prime }$
 1:Obtain the initial weighting matrix Λ(I) 2:Obtain the forward noisy observation x(0),⋯,x(T) using DDIM inversion over the source image I 3:Initialize y(T)←x(T) 4:**for** t=T to $T_{s}$ **do** 5:      Calculate the variance λ(t) corresponding to the time step *t* using Equation (8) 6:      Obtain M(t) using Λ(I) and λ(t) by Equation (13) 7:      Obtain the denoised result H(y(t+Δt),t+Δt) by Equation (4) and guidance scale *w* 8:      Update y(t) by y(t)←M(t)⊙H(y(t+Δt),t+Δt)+(1−M(t))⊙x(t) 9:      **if** $t>T_{s}$ **then**10:            Obtain the denoised result $y^{\prime }\left(t\right)$ by Equation (4) and guidance scale $w^{\prime }$11:            Optimize the text embeddings using $L_{\mathrm{edit}}$ defined in Equation (17)12:      **else**13:            Optimize the weighting matrix using $L_{\mathrm{faithful}}$ defined in Equation (18)14:      **end if**15:**end for**
**Output:** Generated image y conditioned on the source image I


## 5. Experiment

### 5.1. Experimental Setup

**Datasets.** As summarized in Table A1, we evaluate the performance of our method on four diverse datasets, including PIE [18], ZONE [46], Imagen [47] and EMU [48]. The PIE [18] dataset consists of 700 images spanning 10 different editing types, including “objects change”, “attributes change”, “add”, “remove”, etc. The images are categorized into four content groups: animals, humans, indoor scenes, and outdoor scenes. The PIE dataset provides a diverse and challenging set of tasks for evaluating editing capabilities. The ZONE [46] dataset contains 100 samples, including 60 real images sourced from the internet and 40 synthetic images. This dataset focuses on three primary editing operations: 32 images for “add”, 54 images for “change”, and 14 images for “remove”. The Imagen dataset is a synthetic dataset that contains 180 images collected from https://imagen.research.google/ (accessed on 10 April 2025), which are generated by the method of Imagen [47]. Editing prompts are constructed by replacing parts of the original text. Each image undergoes 10 different attribute replacements, resulting in a total of 1800 test examples derived from the original 180 images. The EMU [48] dataset is a more extensive dataset containing 3314 test images across seven categories of image-editing tasks. To ensure high-quality test samples, EMU Edit applies a post-validation phase to filter out low-quality examples. We collect images from the high-quality HQ-Edit dataset [49] to form a new benchmark referred to as the HC dataset. The HC dataset contains 958 images, each with the height and width larger than 1024. During optimization and evaluation, all images are resized to a resolution of 1024 × 1024. These datasets comprehensively cover diverse images and editing tasks, ensuring a robust evaluation of our method.

**Implementation details.** We utilize the pre-trained Stable Diffusion v1.4 as the base model to implement our method. For the HC dataset, we conduct experiments on NVIDIA H20 GPUs equipped with 96 GB memory. All other experiments are conducted on a single NVIDIA GeForce RTX 4090 GPU and using PyTorch framework. For the denoising process, we adopt the DDIM sampler with 50 sampling steps, and we set $T_{s}=25$. We set the guidance scale *w* to 7.5 and $w^{\prime }=2w$. We set the coefficient ρ=0.01. We can adjust the hyperparameters *a* and *b* to flexibly balance the editability and faithfulness. For all qualitative comparisons presented in this paper, we set a=10.0 and b=5.0. These values are empirically chosen to achieve robust performance across various editing tasks. We conduct additional analysis to investigate the effect of different values of hyperparameters in the experimental results. During optimization, to accelerate convergence and stabilize the optimization process, we employ the AdamW optimizer with the learning rate initialized to 0.1 and decaying linearly over time steps.

**Evaluation metrics.** To comprehensively evaluate our method, we employ multiple quantitative metrics, following the evaluation setup of PnP Inversion [18]. These metrics include Structure Distance, PSNR, LPIPS, MSE, SSIM, and CLIP Score, assessing different aspects of editing performance. Structure Distance is computed using deep spatial features extracted by DINO-ViT [50], which measures structural similarity between the edited image and the source image. PSNR, LPIPS, MSE, and SSIM evaluate the content similarity between the edited image and the source image. PSNR, MSE, and SSIM focus on pixel-level similarity, while LPIPS assesses perceptual differences using deep feature representations. The CLIP Score measures text–image consistency by evaluating the cosine similarity between the edited image and the corresponding target prompt using the CLIP model [44]. Unlike PnP Inversion, which computes metrics only for specific regions of the source and edited images, we calculate metrics across the entire images. We argue that evaluating faithfulness should consider the edited regions alongside non-edited regions. For example, when editing a cat into a dog, the faithfulness of the edited image improves if the edited dog inherits certain characteristics of the source cat, such as similar fur color or posture, while maintaining semantic consistency with the target prompt.

### 5.2. Results

In this section, we comprehensively evaluate our method on different datasets. We compare our method with other state-of-the-art image-editing methods, including P2P [2], DiffEdit [4], InstructPix2Pix [51], MasaCtrl [21], NMG [19], PnP Inversion [18], ZONE [46], FPE [22], InfEdit [52], CDS [53], iCD [23], and NGDM [24].

#### 5.2.1. Qualitative Results

**Figure 3:**
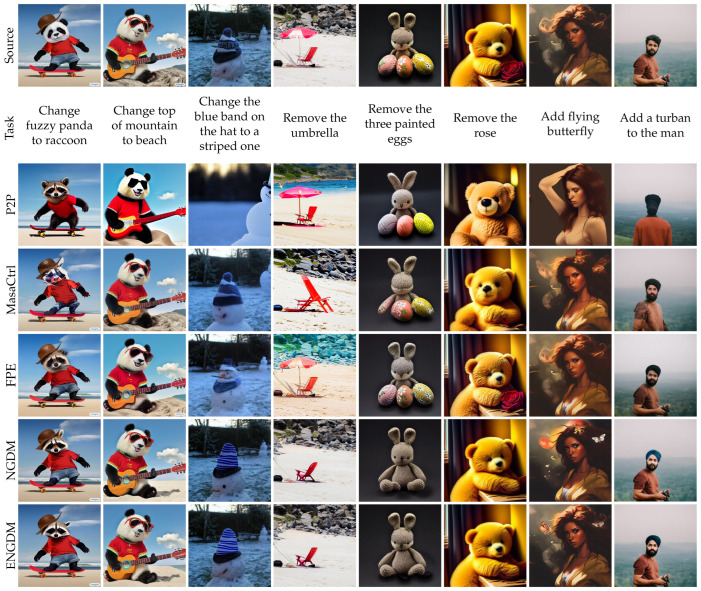
Qualitative comparison with attention-based methods.

**Figure 4:**
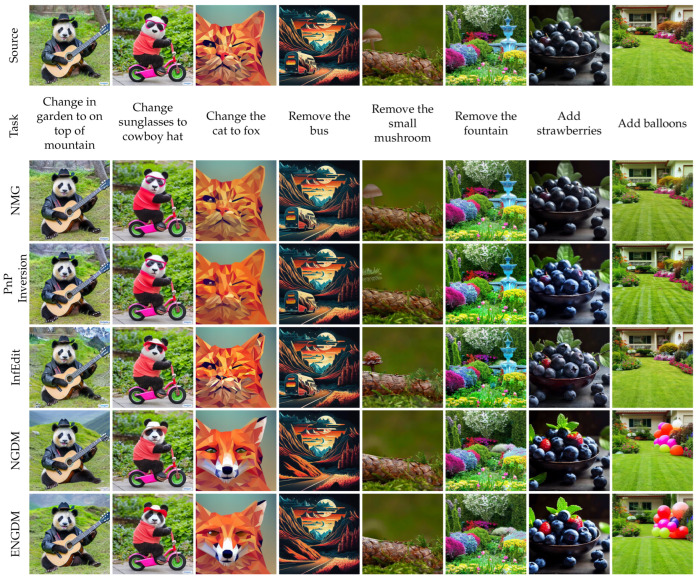
Qualitative comparison with inversion-based methods.

**Figure 5:**
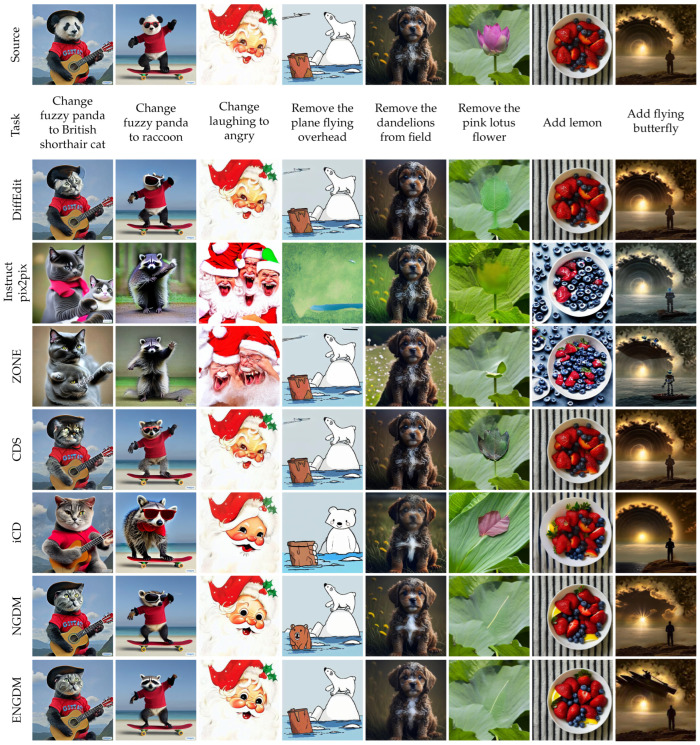
Qualitative comparison with mask-based methods and other methods.

**Figure 6:**
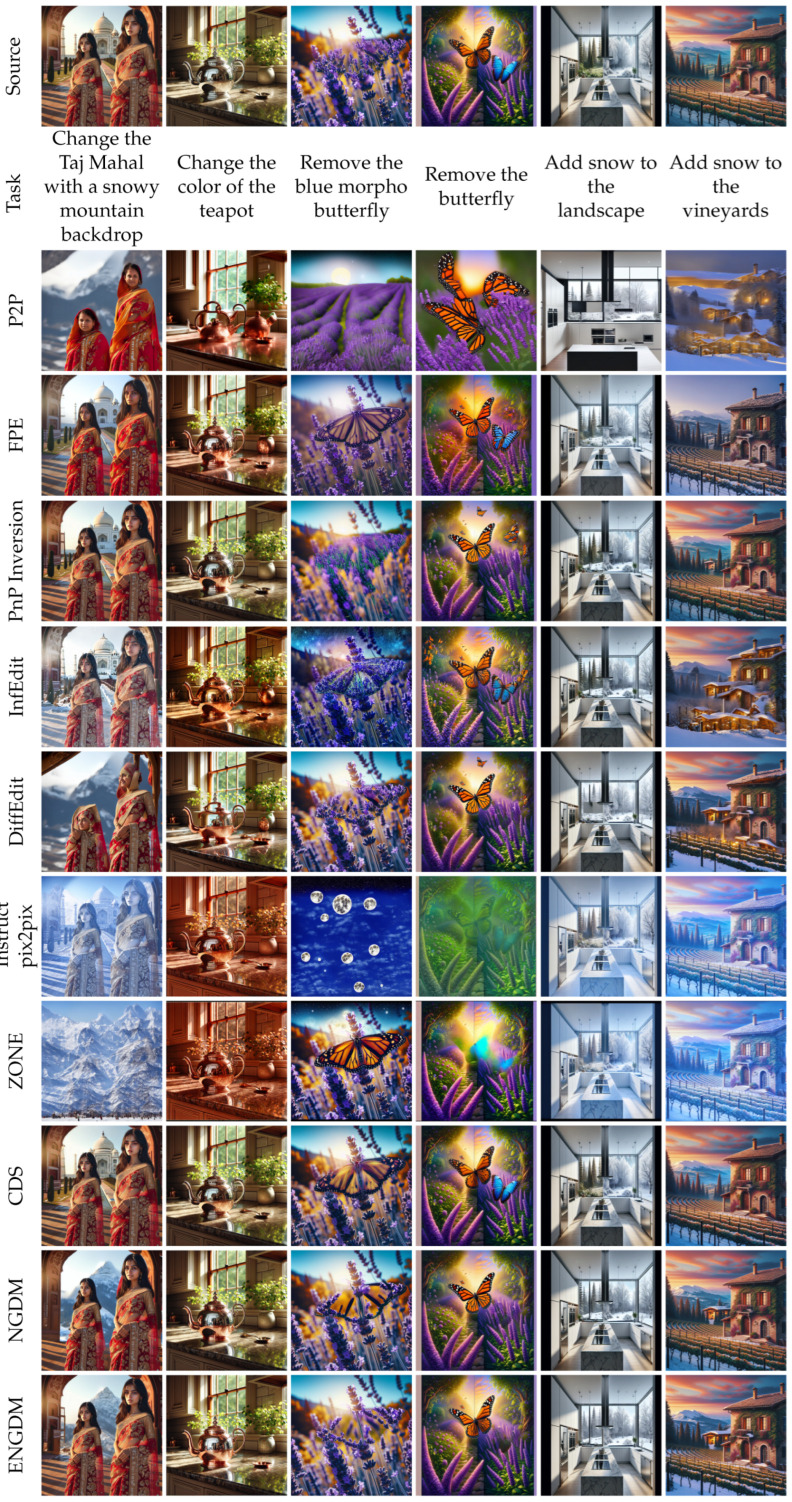
Qualitative comparison on the HC dataset.

Figure 3, Figure 4, Figure 5 and Figure 6 show qualitative comparisons between our method ENGDM and the other baseline methods. In each figure, the first row shows the source image, the second row displays the editing task, and the subsequent rows show the edited images generated by different baseline methods, NGDM, and ENGDM.

Attention-based methods, which manipulate the attention maps in the U-Net network for editing, struggle to precisely control the editing regions. From Figure 3, both P2P and MasaCtrl often lose details in regions that do not require editing, while FPE often fails to achieve the desired edits. For example, in the sixth column, P2P and MasaCtrl successfully perform editing but fail to preserve the original structure of the image, whereas FPE fails to remove the rose, resulting in unsuccessful editing. In contrast, NGDM and ENGDM exhibit better performance. ENGDM further enhances both the faithfulness and editability of NGDM. For instance, in the second column, ENGDM generates a sea that appears more beach-like, while in the fifth column, ENGDM preserves the white cheeks of the original rabbit, retaining finer details of the source image.

Inversion-based methods typically invert the source image into a noisy latent space for editing, but these methods are prone to cumulative errors, leading to editing failures in complex tasks. For instance, in columns 3, 4, 6, 7, and 8 of Figure 4, several inversion-based methods fail to achieve the desired modifications. In contrast, NGDM and ENGDM successfully perform the editing tasks. For the editing tasks in columns 2 and 3, ENGDM outperforms NGDM in maintaining structural consistency with the source image.

Mask-based methods, such as DiffEdit, restrict the editing regions using user-provided or automatically generated masks but often produce edge artifacts. For example, in the first column of Figure 5, DiffEdit generates unnatural artifacts at the edges of the hat, while in the sixth column, the edited flower region appears inconsistent with the rest of the image. Additionally, methods like ZONE and iCD struggle to maintain consistency with the source image in most cases, and CDS fails to perform the editing tasks in columns 4, 5, 7, and 8. In comparison, ENGDM strikes a better balance between consistency with the source image and editability. In addition, as shown in Figure 6, we can see that ENGDM demonstrates superior performance even on the more challenging HC dataset.

#### 5.2.2. Quantitative Results

In this section, we evaluate the performance of ENGDM on four datasets using multiple quantitative metrics and compare ENGDM with multiple baseline methods. We use Structure Distance, PSNR, LPIPS, MSE, and SSIM to assess faithfulness, and the editing score calculated by the CLIP model to assess editability. NGDM and ENGDM allow flexible control of the editability and faithfulness by adjusting the hyperparameters *a* and *b*. To better compare with baseline methods, we provide results for two different versions of ENGDM.

From Table 2, Table 3, Table 4, Table 5 and Table 6, we observe that when a=10.0 and b=5.0, ENGDM achieves the highest editing score across all datasets, with values of 25.97, 25.43, 34.45, 25.75, and 27.63, respectively. As shown in Table 2, Table 3, Table 4 and Table 5, ENGDM significantly outperforms iCD in terms of faithfulness. For example, from Table 2, it can be seen that on the PIE dataset, ENGDM outperforms iCD in terms of Structure Distance/PSNR/LPIPS/MSE/SSIM, with values of 18.80/19.94/146.45/132.19/71.18, compared to 39.43/17.81/235.93/203.94/62.39 for iCD, demonstrating the better faithfulness of ENGDM. As shown in Table 2, Table 3, Table 4, Table 5 and Table 6, compared to NGDM, ENGDM shows further improvements in both the editing score and all evaluation metrics for faithfulness. For example, from Table 2, it can be seen that on the PIE dataset, ENGDM achieves an editing score of 25.97, which is higher than that of NGDM at 25.84. And the Structure Distance/PSNR/LPIPS/MSE/SSIM values of ENGDM are 18.80/19.94/146.45/132.19/71.18, which outperform those of NGDM at 21.32/19.31/159.84/139.30/69.37.

When a=10.0,b=10.0, as shown in Table 2, Table 3 and Table 4 and Table 6, ENGDM achieves the best Structure Distance/PSNR/LPIPS/MSE/SSIM values on the PIE, ZONE, Imagen, and HC datasets, demonstrating the best faithfulness. As shown in Table 5, on the EMU dataset, the Structure Distance/PSNR/LPIPS/MSE/SSIM values of ENGDM are comparable to those of CDS, but ENGDM achieves an editing score of 23.41, outperforming CDS of 22.71. ENGDM outperforms NGDM on all datasets. For example, from Table 2, it can be seen that ENGDM achieves an editing score of 24.92 on the PIE dataset, higher than that of NGDM at 24.65. Furthermore, in terms of faithfulness, the Structure Distance/PSNR/LPIPS/MSE/SSIM values of ENGDM are 6.55/23.98/74.64/48.96/79.84/24.92, which outperform those of NGDM at 7.35/23.40/82.94/57.03/76.08. Overall, combining both the qualitative and quantitative results, ENGDM achieves a better balance between editability and faithfulness compared to NGDM and other baseline methods, resulting in higher-quality edited images.

#### 5.2.3. User Study

We conduct a user study to evaluate the performance of ENGDM on the datasets. We query 40 participants, and each participant is provided with 40 randomly selected source images along with the corresponding editing results generated by different methods. The images generated by our method and the comparison methods are displayed in random order to the participants. Participants are suggested to select the image that best apply the requested editing while preserving the most details of the original image. The percentage of votes for our method compared to the other methods are shown in Table 7, which demonstrates that the participants exhibit a strong preference towards our method.

### 5.3. Ablation Study

#### 5.3.1. Ablation Analysis of ENGDM

**Figure 7:**
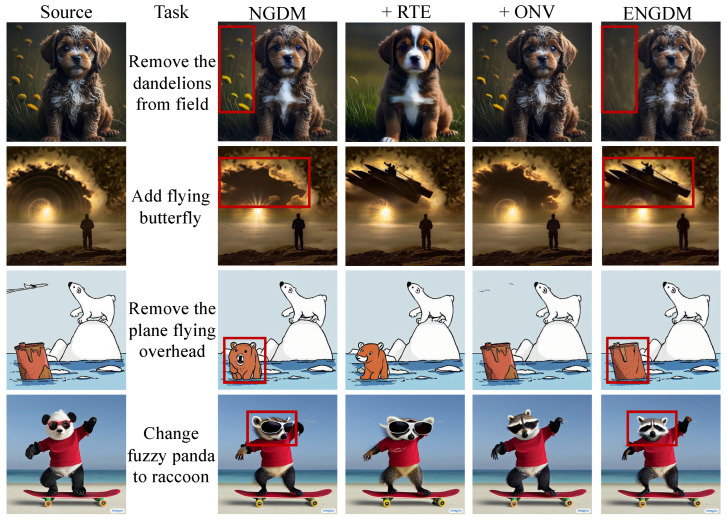
Ablation study of ENGDM. The first column shows the source images, the second column describes the editing tasks, and the third column displays the results generated by NGDM. The fourth/fifth columns show the results obtained by adding only the RTE/ONV technique to NGDM. The final column presents the complete ENGDM, incorporating both RTE and ONV. Red boxes highlight the improvements of ENGDM over NGDM.

In this section, we conduct an ablation study to evaluate the contributions of the two key techniques introduced to NGDM: reinforced text embeddings (RTEs) and optimized noise variances (ONVs). Figure 7 illustrates the results of this ablation study, showcasing the impact of each technique on image-editing performance. RTE enhances the ability of the model to align the generated image with the target prompt by reinforcing the text embeddings during the denoising process. As illustrated in the fourth column of Figure 7, incorporating RTE into NGDM significantly improves editability. For example, in the first row, the result with RTE successfully removes the dandelions compared to NGDM. ONV, on the other hand, focuses on improving the preservation of the source image details by dynamically adjusting the noise variances during the denoising process. As seen in the fifth column, ONV improves the faithfulness of the generated image. For instance, in the third row, the result with ONV better preserves the shape of the wooden barrel. ENGDM, which combines both RTE and ONV, enhances both editability and faithfulness.

#### 5.3.2. Effect of Hyperparameters *a* and *b*

As mentioned in Section 4.4, we transform the attention map A(I) into weighting matrix Γ(I) with hyperparameters *a* and *b*. We control the initial time step of denoising each pixel by adjusting hyperparameters *a* and *b*. To investigate the impact of *a* and *b* on the performance of ENGDM, we conduct the ablation study on the ZONE dataset. We perform experiments by varying *a* and *b* while fixing one of them.

As shown in Table 8, when b=5.0 and *a* is varied, we observe that increasing *a* improves editability at the cost of reduced faithfulness. For instance, ENGDM achieves Structure Distance/PSNR/LPIPS/MSE/SSIM values of 6.01/26.05/55.63/36.26/82.43 when a=6.0, indicating strong faithfulness. However, ENGDM achieves Structure Distance/PSNR/LPIPS/MSE/SSIM values of 27.09/18.14/169.52/190.53/70.08 as *a* increases to 14.0, reflecting a decline in faithfulness. Meanwhile, the CLIP Score increases from 23.89 to 25.61, demonstrating improved editability. This trend suggests that higher values of *a* enhance the ability of the model to align with the target prompt but may compromise the preservation of the source image details.

Conversely, when a=10.0 and *b* is varied, increasing *b* improves faithfulness while reducing editability. For example, when b=3.0, ENGDM achieves a CLIP Score of 25.68, indicating strong editability, but the faithfulness is reduced. As *b* increases to 7.0, the Structure Distance decreases to 6.78, and the PSNR improves to 23.76, indicating enhanced faithfulness. However, the CLIP Score decreases to 24.56, suggesting reduction in editability.

Overall, these results highlight that there is a trade-off between editability and fidelity that can be controlled by tuning hyperparameters *a* and *b*. By adjusting these parameters, ENGDM offers flexibility in balancing the preservation of the source image and the effectiveness of the edits.

#### 5.3.3. Comparison with Hard Weighting Matrix

**Figure 8:**
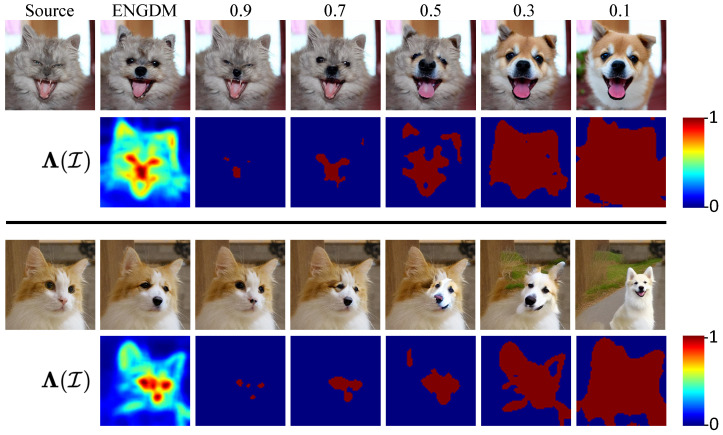
Edited images and heatmaps with soft and hard weighting matrices. The images in the second column represent the results generated by our method and the heatmap below the image depicts the weighting matrix Λ(I) defined in Section 4.1 in the paper. A color bar is presented in the left to illustrate the corresponding colors for different values. The images in columns 3–7 represent the results generated using the hard weighting matrix with threshold value in {0.1,0.3,0.5,0.7,0.9} obtained by Λ(I)=I(A(I)≥η), where η is a threshold chosen in {0.1,0.3,0.5,0.7,0.9}. The heatmaps below the images represent the binary hard weighting matrix.

To further analyze the effectiveness of the soft weighting matrix Λ(I) used in ENGDM, we compare the results with the method using the hard weighting matrix applied at different threshold values. As shown in Figure 8, the first column displays the source image, and the second column shows the results generated by ENGDM with the soft weighting matrix, along with the corresponding heatmap. The heatmap visually represents the weighting values, where brighter regions correspond to higher noise variance, which indicates greater editability, while darker regions correspond to lower noise variance, indicating greater faithfulness to the original image. In contrast, columns 3 to 7 present the results generated using hard weighting matrices with threshold of 0.1, 0.3, 0.5, 0.7, and 0.9.

During the denoising process, methods guided by a hard mask consistently edit regions exceeding the threshold. ENGDM utilizes soft weights to gradually denoise each pixel based on its required degree of editing. As shown in Figure 8, hard mask-guided methods often produce artifacts at the edges of the mask, leading to unnatural transitions between edited and non-edited regions. Additionally, hard mask-guided methods tend to over-edit the targeted regions, reducing faithfulness to the source image. In comparison, ENGDM generates more natural images without the edge artifacts, effectively achieving the desired edits while maintaining high faithfulness to the source image.

#### 5.3.4. Results at the Intermediate Steps of the Forward and Reverse Process

**Figure 9:**
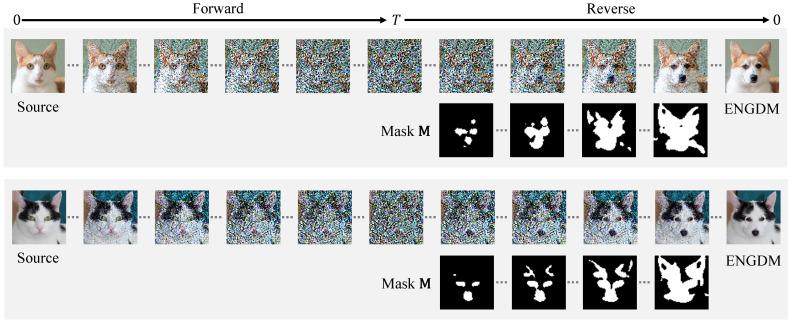
Results at the intermediate steps of the forward process and reverse process. We present the results at the intermediate steps of the forward and reverse process, along with the mask M at the intermediate steps during the denoising, which is defined in Equation (13). The white regions in the mask image indicate the pixels that have undergone the denoising process, while the black regions represent the pixels that have not yet been denoised.

Figure 9 presents the evolution of the image during the forward and reverse processes when editing a cat into a dog, along with the masks M used at each step of the reverse process as described in Algorithm 1. The mask M dynamically indicates which pixels have undergone denoising (black regions) and which pixels remain unprocessed (white regions). At the early stages of the reverse process, ENGDM prioritizes denoising the regions that require more editing, such as the prominent features of the cat, including the eyes and nose as indicated by the white areas in the mask. As the process progresses, denoising extends to the facial region of the cat and eventually to the background. This reflects the ability of ENGDM to gradually denoise different pixels based on their required editing degree. The progressive denoising method enables ENGDM to achieve precise effective editing while better preserving the details of the original image.

#### 5.3.5. Validation of the Method for Determining the Total Diffusion Time Step

**Figure 10:**
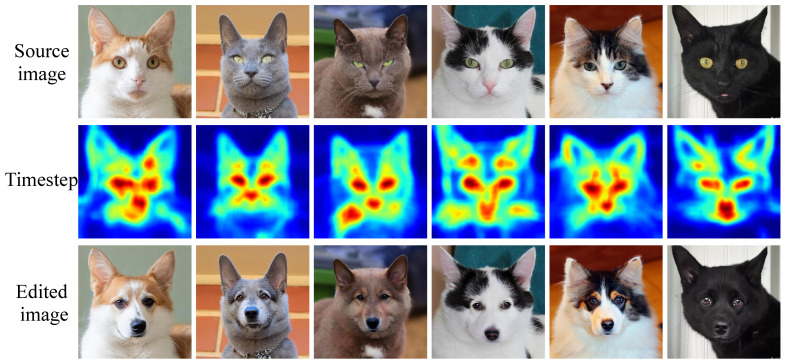
Visualization of the total diffusion time step for each pixel. The first row shows the source images. The second row visualizes the total diffusion time step of each pixel, where brighter colors indicate a larger total diffusion time step. The third row presents the edited images generated under the editing task of editing a cat into a dog.

To validate the effectiveness of this method, we visualize the determined total diffusion time steps in Figure 10 of the response letter. As can be seen, when editing a cat into a dog, the prominent features of the cat, such as the eyes and nose, are assigned larger total diffusion time steps, whereas the background is assigned smaller total diffusion time steps. This demonstrates that our method can successfully assign the total diffusion time step for each pixel based on the degree of required editing. Moreover, as seen in the edited image in the third row of Figure 10, our method effectively edits the cat into a dog while preserving similarity to the source image, which further validates the effectiveness of the method. In addition, we also experiment with a random strategy for assigning total diffusion time to each pixel. Table 9 presents a quantitative comparison of the two approaches on the PIE dataset. The results show that our method achieves a better balance, while the random method significantly decreases the editing score.

## 6. Conclusions

In this paper, we propose ENGDM for progressive image editing. ENGDM is constructed by adding independent Gaussian noises with varying variances to different image pixels. To avoid retraining, we rectify ENGDM by assigning different total diffusion times to different pixels, thereby implementing progressive editing within an isotropic framework. Furthermore, we enhance editability by leveraging reinforced text embeddings and improve faithfulness through optimization of noise variances. Extensive experimental results demonstrate the effectiveness of ENGDM across multiple datasets. While the optimization procedure brings significant performance improvements, it also introduces computational overhead. Furthermore, the performance of ENGDM relies on the underlying diffusion model. Failures in the generation process of the underlying model can lead to failures in the editing results. In the future, we plan to extend ENGDM to more advanced diffusion models and explore its applications in broader domains such as video editing.

## Figures and Tables

**Table 2:** Quantitative comparisons in image editing. Evaluated using the PIE dataset. Different metrics are scaled. The best results are highlighted in **bold** while the second best results are marked with an underline. The arrow pointing upwards indicates that a larger value of the metric is better, while the arrow pointing downwards indicates that a smaller value of the metric is better.

Method	Structure	Content Preservation				Editing
	**Distance** ↓	**PSNR** ↑	**LPIPS** ↓	**MSE** ↓	**SSIM** ↑	**Score** ↑
P2P [2]	69.95	15.10	335.58	347.18	55.36	24.98
DiffEdit [4]	17.41	19.66	129.14	131.34	72.43	25.09
InstructPix2Pix [51]	57.94	16.71	269.02	419.30	61.72	23.57
MasaCtrl [21]	28.08	19.09	181.21	147.28	67.72	23.90
NMG [19]	15.37	23.39	112.16	160.12	73.48	23.57
PnP Inversion [18]	11.71	22.26	116.40	76.16	73.29	24.84
ZONE [46]	58.27	16.20	281.79	396.66	58.97	23.98
FPE [22]	12.77	21.67	114.95	82.86	73.42	24.35
InfEdit [52]	19.47	21.49	133.87	176.39	70.78	24.74
CDS [53]	7.33	23.83	76.48	57.27	76.79	23.91
iCD [23]	39.43	17.81	235.93	203.94	62.39	25.92
**NGDM (a = 10.0, b = 5.0)**	21.32	19.31	159.84	139.30	69.37	25.84
**ENGDM (a = 10.0, b = 5.0)**	18.80	19.94	146.45	132.19	71.18	**25.97**
**NGDM (a = 10.0, b = 10.0)**	7.35	23.40	82.94	57.03	76.08	24.65
**ENGDM (a = 10.0, b = 10.0)**	**6.55**	**23.98**	**74.64**	**48.96**	**79.84**	24.92

**Table 3:** Quantitative comparisons in image-editing tasks. Evaluated using the ZONE dataset. Different metrics are scaled. The best results are highlighted in **bold** while the second best results are marked with an underline. The arrow pointing upwards indicates that a larger value of the metric is better, while the arrow pointing downwards indicates that a smaller value of the metric is better.

Method	Structure	Content Preservation				Editing
	**Distance** ↓	**PSNR** ↑	**LPIPS** ↓	**MSE** ↓	**SSIM** ↑	**Score** ↑
P2P [2]	57.96	16.00	265.69	286.01	59.92	24.19
DiffEdit [4]	14.87	20.39	104.78	117.71	77.29	24.94
InstructPix2Pix [51]	33.86	18.70	189.72	296.25	69.99	24.19
MasaCtrl [21]	24.20	19.78	151.36	133.62	72.98	23.71
NMG [19]	15.95	23.61	95.19	105.59	78.23	23.01
PnP Inversion [18]	11.36	22.49	94.35	74.29	77.91	24.20
ZONE [46]	34.60	17.58	204.14	295.32	67.89	24.64
FPE [22]	11.41	22.37	90.11	74.02	78.30	23.48
InfEdit [52]	15.74	21.69	106.04	152.23	74.21	24.18
CDS [53]	6.91	24.49	63.52	57.11	81.79	23.72
iCD [23]	32.85	17.76	205.44	198.61	64.50	25.23
**NGDM (a = 10.0, b = 5.0)**	17.93	19.97	130.55	124.57	74.11	25.12
**ENGDM (a = 10.0, b = 5.0)**	16.75	20.17	122.75	119.58	74.73	**25.43**
**NGDM (a = 10.0, b = 10.0)**	6.27	24.14	64.57	48.00	81.44	23.95
**ENGDM (a = 10.0, b = 10.0)**	**5.64**	**24.69**	**57.84**	**41.36**	**83.82**	24.36

**Table 4:** Quantitative comparisons in image-editing tasks. Evaluated using the Imagen dataset. Different metrics are scaled. The best results are highlighted in **bold** while the second best results are marked with an underline. The arrow pointing upwards indicates that a larger value of the metric is better, while the arrow pointing downwards indicates that a smaller value of the metric is better.

Method	Structure	Content Preservation				Editing
	**Distance** ↓	**PSNR** ↑	**LPIPS** ↓	**MSE** ↓	**SSIM** ↑	**Score** ↑
P2P [2]	56.38	14.07	284.17	405.39	56.55	32.99
DiffEdit [4]	13.05	19.54	92.93	121.29	78.51	33.60
InstructPix2Pix [51]	59.35	12.66	336.20	690.25	56.59	25.89
MasaCtrl [21]	20.40	18.27	158.14	160.87	72.01	32.62
NMG [19]	7.87	22.04	80.81	76.93	78.63	32.82
PnP Inversion [18]	8.95	21.12	82.39	87.80	77.89	33.13
ZONE [46]	60.35	12.67	344.80	617.58	55.23	25.28
FPE [22]	9.18	20.56	85.44	93.33	76.62	32.78
InfEdit [52]	6.55	22.28	66.20	70.64	80.92	32.66
CDS [53]	8.95	20.44	75.63	111.85	78.73	32.87
iCD [23]	31.42	16.49	212.42	242.45	66.02	34.33
**NGDM (a = 10.0, b = 5.0)**	15.73	18.89	121.07	138.81	75.12	34.23
**ENGDM (a = 10.0, b = 5.0)**	14.40	19.31	110.98	126.34	76.20	**34.45**
**NGDM (a = 10.0, b = 10.0)**	5.80	22.38	61.33	62.00	81.06	33.24
**ENGDM (a = 10.0, b = 10.0)**	**5.01**	**22.99**	**54.82**	**55.23**	**81.74**	33.46

**Table 5:** Quantitative comparisons in image-editing tasks. Evaluated using the EMU dataset. Different metrics are scaled. The best results are highlighted in **bold** while the second best results are marked with an underline. The arrow pointing upwards indicates that a larger value of the metric is better, while the arrow pointing downwards indicates that a smaller value of the metric is better.

Method	Structure	Content Preservation				Editing
	**Distance** ↓	**PSNR** ↑	**LPIPS** ↓	**MSE** ↓	**SSIM** ↑	**Score** ↑
P2P [2]	79.14	14.02	393.78	434.62	46.02	24.97
DiffEdit [4]	17.68	20.28	124.93	128.29	70.51	23.73
InstructPix2Pix [51]	48.68	17.45	236.15	353.48	61.62	22.49
MasaCtrl [21]	31.30	18.66	200.59	161.27	63.76	23.18
NMG [19]	11.15	**26.69**	78.58	73.74	76.55	21.88
PnP Inversion [18]	16.16	21.18	154.09	89.96	68.52	25.54
ZONE [46]	52.22	16.44	264.65	377.38	58.02	22.70
FPE [22]	12.25	21.97	113.57	77.49	72.15	23.91
InfEdit [52]	36.59	16.92	226.86	260.86	57.44	25.09
CDS [53]	**5.53**	25.48	**59.32**	**38.36**	**77.12**	22.71
iCD [23]	53.94	16.25	305.91	266.68	52.17	25.71
**NGDM (a = 10.0, b = 5.0)**	31.94	18.60	210.35	191.05	62.33	25.58
**ENGDM (a = 10.0, b = 5.0)**	27.61	19.16	189.40	165.95	64.26	**25.75**
**NGDM (a = 10.0, b = 10.0)**	7.82	23.93	78.91	51.25	75.07	23.20
**ENGDM (a = 10.0, b = 10.0)**	6.15	24.67	69.50	43.93	76.14	23.41

**Table 6:** Quantitative comparisons in image-editing tasks. Evaluated using the HC dataset. Different metrics are scaled. The best results are highlighted in **bold** while the second best results are marked with an underline. The arrow pointing upwards indicates that a larger value of the metric is better, while the arrow pointing downwards indicates that a smaller value of the metric is better.

Method	Structure	Content Preservation				Editing
	**Distance** ↓	**PSNR** ↑	**LPIPS** ↓	**MSE** ↓	**SSIM** ↑	**Score** ↑
P2P [2]	90.84	11.65	427.75	779.03	30.55	24.63
DiffEdit [4]	9.43	20.24	77.34	111.52	76.30	26.94
InstructPix2Pix [51]	67.04	13.82	283.82	569.97	42.53	25.01
PnP Inversion [18]	8.68	22.82	69.11	77.97	77.35	26.24
ZONE [46]	68.14	13.40	334.81	594.43	43.51	24.77
FPE [22]	20.10	18.84	135.29	150.59	74.47	26.31
InfEdit [52]	31.34	16.42	191.28	300.09	63.67	26.37
CDS [53]	3.38	28.38	34.61	27.36	90.83	25.79
**NGDM (a = 10.0, b = 5.0)**	12.10	19.54	106.02	122.53	72.88	27.36
**ENGDM (a = 10.0, b = 5.0)**	11.16	19.95	97.95	117.11	73.70	**27.63**
**NGDM (a = 10.0, b = 10.0)**	3.91	30.20	34.84	27.03	90.27	25.94
**ENGDM (a = 10.0, b = 10.0)**	**3.02**	**30.72**	**30.35**	**22.62**	**92.84**	26.27

**Table 7:** User study results on the evaluation datasets. The best results are highlighted in **bold** while the second best results are marked with an underline.

P2P	DiffEdit	InstructPix2Pix	MasaCtrl	NMG	PnP Inversion	ZONE	FPE	InfEdit	CDS	iCD	NGDM	ENGDM
0.63%	10.31%	3.75%	2.19%	3.13%	1.88%	6.88%	1.88%	5.00%	6.56%	9.06%	19.06%	**29.69%**

**Table 8:** The performance on the ZONE dataset with varying values of *a* or *b* while respectively fixing b=5.0 or a=10.0. The arrow pointing upwards indicates that a larger value of the metric is better, while the arrow pointing downwards indicates that a smaller value of the metric is better.

*a* (b=5.0)	6.0	8.0	10.0	12.0	14.0
Distance ↓	6.01	10.14	16.75	22.70	27.09
PSNR ↑	26.05	22.18	20.17	18.89	18.14
LPIPS ↓	55.63	88.41	122.75	150.47	169.52
MSE ↓	36.26	73.49	119.58	160.75	190.53
SSIM ↑	82.43	78.42	74.73	72.02	70.08
Score ↑	23.89	24.73	25.43	25.56	25.61
b **(**a=10.0**)**	**3.0**	**4.0**	**5.0**	**6.0**	**7.0**
Distance ↓	34.07	24.45	16.75	10.66	6.78
PSNR ↑	17.50	18.67	20.17	21.85	23.76
LPIPS ↓	196.95	160.33	122.75	90.14	66.39
MSE ↓	216.30	168.29	119.58	79.52	51.23
SSIM ↑	67.12	70.91	74.73	78.30	81.13
Score ↑	25.68	25.64	25.43	24.82	24.56

**Table 9:** Quantitative comparison results of our and random methods. Evaluated using the ZONE dataset. Different metrics are scaled. The arrow pointing upwards indicates that a larger value of the metric is better, while the arrow pointing downwards indicates that a smaller value of the metric is better.

Method	Structure	Content Preservation				Editing
	**Distance** ↓	**PSNR** ↑	**LPIPS** ↓	**MSE** ↓	**SSIM** ↑	**Score** ↑
Random	13.83	21.66	114.95	109.81	75.16	23.96
Ours	16.75	20.17	122.75	119.58	74.73	25.43

## Data Availability

Data are available upon request.

## Appendix A. Mathematical Definitions of Evaluation Metrics

To comprehensively evaluate our method, we employ multiple quantitative metrics, following the evaluation setup of PnP Inversion [18]. These metrics include Structure Distance, PSNR, LPIPS, MSE, SSIM, and CLIP Score, assessing different aspects of editing performance. Let x,y,C denote the source image, edited image, the source prompt and the target prompt, respectively; the mathematical definitions of these metrics are as follows.

(1) **Structure Distance (SD)** utilizes spatial features extracted by DINO-ViT [50] to compute structural alignment:
  $$\mathrm{SD}=∥f_{\mathrm{DINO}}\left(x\right)-f_{\mathrm{DINO}}{\left(y\right)∥}_{2}^{2},$$
  where $f_{\mathrm{DINO}}$ represents the deep spatial features extracted from the input image using the DINO model.
(2) **Peak Signal-to-Noise Ratio (PSNR)** quantifies the maximum signal-to-noise ratio based on the mean squared error:
  $$\mathrm{PSNR}=10\cdot \log_{10}\left(\frac{{\mathrm{MAX}}_{I}^{2}}{\mathrm{MSE}(x,y)}\right),$$
  where ${\mathrm{MAX}}_{I}$ is the maximum possible pixel value and MSE is the mean squared error.
(3) **Learned Perceptual Image Patch Similarity (LPIPS)** assesses perceptual differences using deep feature representations:
  $$\mathrm{LPIPS}=\sum_{l}w_{l}\cdot {∥f_{l}\left(x\right)-f_{l}\left(y\right)∥}_{2}^{2},$$
  where $f_{l}$ denotes the features from layer *l* of a pre-trained network and $w_{l}$ is the learned weights for the *l*-th layer.
(4) **Mean Squared Error (MSE)** evaluates pixel-level reconstruction quality by averaging the squared differences between images:
  $$\mathrm{MSE}=\frac{1}{N}\sum_{i=1}^{N}{\left(x_{i}-y_{i}\right)}^{2},$$
  where *N* denotes the total number of pixels and *i* denotes the index of the pixel.
(5) **Structural Similarity Index (SSIM)** measures the structural similarity:
  $$\mathrm{SSIM}=\frac{\left(2\mu_{x}\mu_{y}+C_{1}\right)\left(2\sigma_{xy}+C_{2}\right)}{\left(\mu_{x}^{2}+\mu_{y}^{2}+C_{1}\right)\left(\sigma_{x}^{2}+\sigma_{y}^{2}+C_{2}\right)},$$
  where μ, σ, and $\sigma_{xy}$ denote the mean, standard deviation, and covariance of x and y, respectively. $C_{1}={\left(k_{1}L\right)}^{2}$, $C_{2}={\left(k_{2}L\right)}^{2}$ with *L* is the dynamic range and $k_{1}=0.01$, $k_{2}=0.03$.
(6) **CLIPScore** evaluates text–image alignment via cosine similarity in CLIP space:
  $$\mathrm{CLIPScore}=\frac{f_{I}\left(y\right)\cdot f_{T}\left(C\right)}{∥f_{I}\left(y\right)∥∥f_{T}\left(C\right)∥},$$
  where $f_{I}\left(y\right)$ and $f_{T}\left(C\right)$ are CLIP embeddings of image y and C.

## Appendix B. Table for Describing the Evaluation Dataset

We provide Table A1 to clearly describe all evaluation datasets. This table includes key details such as the dataset name, description, source, size, and editing type.

**Table A1:** Evaluation datasets.

Dataset Name	Description	Source	Size	Editing Type
PIE [18]	PIE dataset grouped into animals, humans, indoor, and outdoor scenes, offering diverse, challenging tasks for evaluating editing performance.	https://github.com/cure-lab/PnPInversion (accessed on 10 April 2025)	700	Add, change, and remove.
ZONE [46]	Zone dataset includes real and synthetic images, focusing on three editing types.	https://drive.google.com/file/d/1lAwpENoDcO1QyFuwz3iKJJ7DmDTFMvIU/view (accessed on 10 April 2025)	100	Add, change, and remove.
Imagen [47]	Imagen dataset contains 180 synthetic images, which are generated by Imagen [47]. Each image is edited with 10 attribute replacements via prompt modification, yielding 1800 evaluation examples.	https://imagen.research.google/ (accessed on 10 April 2025)	1800	Change.
EMU [48]	EMU is a large-scale benchmark, including images spanning seven editing categories. A post-validation step filters out low-quality samples to ensure high data quality.	https://huggingface.co/datasets/facebook/emu_edit_test_set (accessed on 10 April 2025)	3314	Add, change, and remove.
HC	We collect images with the height and width larger than 1024 from the high-quality HQ-Edit dataset [49] to form a new benchmark, which is referred to as the HC dataset.	https://huggingface.co/datasets/UCSC-VLAA/HQ-Edit (accessed on 10 April 2025)		Add, change, and remove.

## Appendix C. Method for Hyperparameters Selection

We propose a systematic approach for hyperparameter selection. The main idea is to iteratively select better parameters based on a balance metric that considers both editability and fidelity. Specifically, let y, $C_{x}$, and $C_{y}$ denote the edited image, source prompt, and target prompt, respectively. We design the balance metric using the CLIP model as follows:

(A1) $$D=S_{t}-S_{s}=\frac{f_{I}\left(y\right)\cdot f_{T}\left(C_{y}\right)}{∥f_{I}\left(y\right)∥∥f_{T}\left(C_{y}\right)∥}-\frac{f_{I}\left(y\right)\cdot f_{T}\left(C_{x}\right)}{∥f_{I}\left(y\right)∥∥f_{T}\left(C_{x}\right)∥}$$

where $f_{I}\left(y\right)$, $f_{T}\left(C_{y}\right)$, and $f_{T}\left(C_{x}\right)$ are CLIP embeddings of y, $C_{x}$, and $C_{y}$, respectively. $S_{t}$ represents the cosine similarity between the edited image and the target prompt, measuring editability. And $S_{s}$ represents the cosine similarity between the edited image and the source prompt, measuring faithfulness. We aim to select parameters that strike a better balance between editability and faithfulness. We start by adjusting *b* within the range [1.0,10.0]. After generating the edited image each time, we compute the balance metric D, and adjust b using b+D. If D>0, b+D increases the value of *b* to enhance faithfulness. If D<0, b+D decreases the value of *b* to enhance editability. If *b* exceeds the range [1.0,10.0] within the maximum iteration count, we proceed to adjust *a* using a−D. The iteration stops when the maximum iteration count is reached or when D=0. We conduct experiments using the proposed parameter selection method (ENGDM-A) on the PIE and ZONE datasets to validate its effectiveness. The results are shown in Table A2. As can be seen, the automatic parameter selection method based on the search metric achieves a better balance with significantly improved faithfulness and a comparable editing score.

**Table A2:** Quantitative comparison results of ENGDM and ENGDM-A methods. Evaluated using the PIE and ZONE datasets. Different metrics are scaled. The arrow pointing upwards indicates that a larger value of the metric is better, while the arrow pointing downwards indicates that a smaller value of the metric is better.

Dataset	Method	Structure	Content Preservation				Editing
		**Distance** ↓	**PSNR** ↑	**LPIPS** ↓	**MSE** ↓	**SSIM** ↑	**Score** ↑
PIE	ENGDM	18.80	19.94	146.45	132.19	71.18	25.87
	ENGDM-A	13.41	21.41	111.77	100.43	73.49	25.82
ZONE	ENGDM	16.75	20.17	122.75	119.58	74.73	25.43
	ENGDM-A	12.64	21.43	100.74	91.38	77.72	25.39
